# Environmental factors associated with the distribution of visceral leishmaniasis in endemic areas of Bangladesh: modeling the ecological niche

**DOI:** 10.1186/s41182-017-0054-9

**Published:** 2017-05-12

**Authors:** Abu Yousuf Md Abdullah, Ashraf Dewan, Md Rakibul Islam Shogib, Md Masudur Rahman, Md Faruk Hossain

**Affiliations:** 1 Department of Geography and Environment, University of Dhaka, University Road, Dhaka, 1000 Bangladesh; 2 Department of Spatial Sciences, Curtin University, Perth, Australia

**Keywords:** Ecological niche model (ENM), Visceral leishmaniasis (VL), Bangladesh, Environmental factors, Human VL cases, Disease modeling

## Abstract

**Background:**

Visceral leishmaniasis (VL) is a parasitic infection (also called kala-azar in South Asia) caused by *Leishmania donovani* that is a considerable threat to public health in the Indian subcontinent, including densely populated Bangladesh. The disease seriously affects the poorest subset of the population in the subcontinent. Despite the fact that the incidence of VL results in significant morbidity and mortality, its environmental determinants are relatively poorly understood, especially in Bangladesh. In this study, we have extracted a number of environmental variables obtained from a range of sources, along with human VL cases collected through several field visits, to model the distribution of disease which may then be used as a surrogate for determining the distribution of *Phlebotomus argentipes* vector, in hyperendemic and endemic areas of Mymensingh and Gazipur districts in Bangladesh. The analysis was carried out within an ecological niche model (ENM) framework using a maxent to explore the ecological requirements of the disease.

**Results:**

The results suggest that VL in the study area can be predicted by precipitation during the warmest quarter of the year, land surface temperature (LST), and normalized difference water index (NDWI). As *P. argentipes* is the single proven vector of *L. donovani* in the study area, its distribution could reasonably be determined by the same environmental variables. The analysis further showed that the majority of VL cases were located in *mauzas* where the estimated probability of the disease occurrence was high. This may reflect the potential distribution of the disease and consequently *P. argentipes* in the study area.

**Conclusions:**

The results of this study are expected to have important implications, particularly in vector control strategies and management of risk associated with this disease. Public health officials can use the results to prioritize their visits in specific areas. Further, the findings can be used as a baseline to model how the distribution of the disease caused by *P. argentipes* might change in the event of climatic and environmental changes that resulted from increased anthropogenic activities in Bangladesh and elsewhere.

## Background

Visceral leishmaniasis (hereafter, VL) caused by *Leishmania donovani* is transmitted to humans by the bite of the sand fly vector. Some of the prominent species include *Phlebotomus argentipes* [1], *Phlebotomus orientalis* in East African lowlands [2], *Phlebotomus papatasi* in Southwest Asia [3], and *Phlebotomus martini* in Kenya/Ethiopia [4]. The disease is endemic in 98 countries with an estimated global burden of 300 million people [5]. Annually, between 20,000 and 40,000 human fatalities are believed to be attributed to this disease worldwide [6], and the risk of insurgence/resurgence or spread into new areas is likely to increase with the changing climate [7].

Of the total global incidences of VL (also called kala-azar in South Asia), more than 67% of the cases are found in the Indian subcontinent, largely affecting the poorest in a population [6, 8]. The literature suggests that the number of people at risk in India, Bangladesh, and Nepal ranges from 200 to 300 million [9], and the annual economic impact is estimated to be US$350 million [10]. Because of the significant increase in VL cases in the Indian subcontinent, in 2005, India, Bangladesh, and Nepal undertook a program to eliminate the disease [11].

Environmental factors acquired from various sources, including geographic information and remotely sensed data, have been used to predict and elucidate the distribution of the disease caused by vectors. This approach has proven to be highly useful for disease prevention and has been used to forecast epidemics, which is imperative for the preparedness of health systems to cope with such outbreaks [3, 4, 7, 12–19]. For example, land surface temperature and vegetation index obtained from satellite data showed significant correlation with the occurrence of sand flies in East Africa [4]. An ecological niche model (hereinafter, ENM) showed that the distribution of the sand fly vector was strongly linked with land cover type in the Middle East [14]. *P. papatasi* was found to be associated with vegetation in Southwest Asia [3]. Using two predictive models, Nieto et al. [20] showed that the highest VL risk in the interior region of Brazil was linked with a semiarid and hot climate, while the coastal forest region was unsuitable for sand fly. Spatiotemporal dynamics of vector species and human dengue cases was investigated using the monthly normalized difference vegetation index from NOAA–AVHRR data together with surface properties and topographic index [17]. Their study revealed significant correspondence between predicted vector activity and human dengue cases in South America. Similarly, elevation, rainfall, temperature, and forest cover were found to be associated with the distribution of sand flies in France [13]. Therefore, Guernier et al. [21] emphasized that consideration of a combination of ecological and climatic factors could greatly enhance the understanding about the distribution of human pathogens. All of the studies noted above have demonstrated the influence of environmental factors on the occurrence of disease and, consequently, potential distribution of vectors in various settings, which could support targeted interventions to tackle vector-borne diseases such as VL caused by sand fly.

Various modeling techniques are now available to integrate environmental layers with disease cases, which allow environmental factors to be isolated and potential vector distribution to be mapped. Among them, the ENM has played a vital role in determining the underlying factors that contribute to the spatial patterns of the disease [17]. It is a powerful tool because of its ability to predict the distribution of vectors in areas where detailed sampling is lacking [22], and has been utilized in various studies around the world to model dengue [23], malaria [24, 25], canine leishmaniasis [13], anthrax [15], visceral leishmaniasis [20, 26], leishmaniasis transmitted by *Lutzomyia* [27–29], Chagas disease [19], and Japanese encephalitis [16]. For example, using climatic variables together with topographic parameters in an ENM, González et al. [7] predicted that climate change will exacerbate the ecological risk of human exposure to leishmaniasis in North America.

Although the incidence of VL is not a new phenomenon in Bangladesh, the major resurgence after the 1990s was mainly attributed to the cessation of DDT (dichloro diphenyl trichloroethane) spraying, originally undertaken to control malaria vectors in South Asia [30, 31]. To date, a number of initiatives were taken into account to eliminate the disease from Bangladesh, of which the VL elimination program is the most recent and currently in place [32]. With the introduction of the program in 2005, there had been a sharp decrease in the number of cases [11, 33]. Environmental vector management was implemented as part of the elimination program, for instance in Mymensingh district, but its effectiveness showed mixed outcomes [34]. Le Rutte et al. [35] emphasized that such measures require additional intervention in highly endemic areas. Thornton et al. [36] emphasized that a “one size fits all” strategy may not be an appropriate approach because VL occurrence is a multifaceted problem. As noted by Joshi et al. [10], complete elimination may be difficult to achieve in South Asia due to a number of reasons, including shortcomings of disease surveillance systems and resource constraints. Because the natural environment is constantly disturbed by humans through agricultural development and/or deforestation due to the ever-increasing population in Bangladesh, minimizing “ecological risk” of VL spread [7] could be an important alternative to keep its occurrence at an acceptable level. The exact terrestrial habitat of the sand fly vector is largely unknown [37]; therefore, modeling the distribution of disease based on environmental conditions could provide the information required for the effective management of this fatal disease [38–40]. The outcome may then guide health officials in making informed decisions and targeted interventions.

Although a number of studies of the clinical manifestations, epidemiological features, and socioeconomic aspect of VL have been conducted in Bangladesh [9, 11, 33, 34, 41–55], very few studies have used VL cases together with environmental variables to investigate the disease occurrence and factors affecting its distribution, particularly in endemic areas of Bangladesh [56]. This study aims to fill this gap with an assumption that if the probability of the distribution of the disease can be modeled, then the occurrence of vectors could be determined since their geographical spread appears to be the same. Here, the human cases obtained from the field visits were used to model the distribution of VL. As sand flies are found to be distributed in and around infected households in endemic areas of Bangladesh [54, 56, 57], we believe that using human VL cases, in the absence of actual vector locations, could provide valuable insights into the likelihood of vectors over space and contribute to the understanding of the geographic ecology of the vectors.

In this work, first, we aimed to develop an ENM to predict the distribution of disease in endemic areas of Bangladesh by assuming that its distribution is likely to follow the incidence of its vectors. Second, we attempted to characterize the environmental and ecological conditions suitable for the occurrence of *P. argentipes*, which is important in formulating appropriate measures for effective health-care delivery.

## Methods

### Study area

**Fig. 1:**
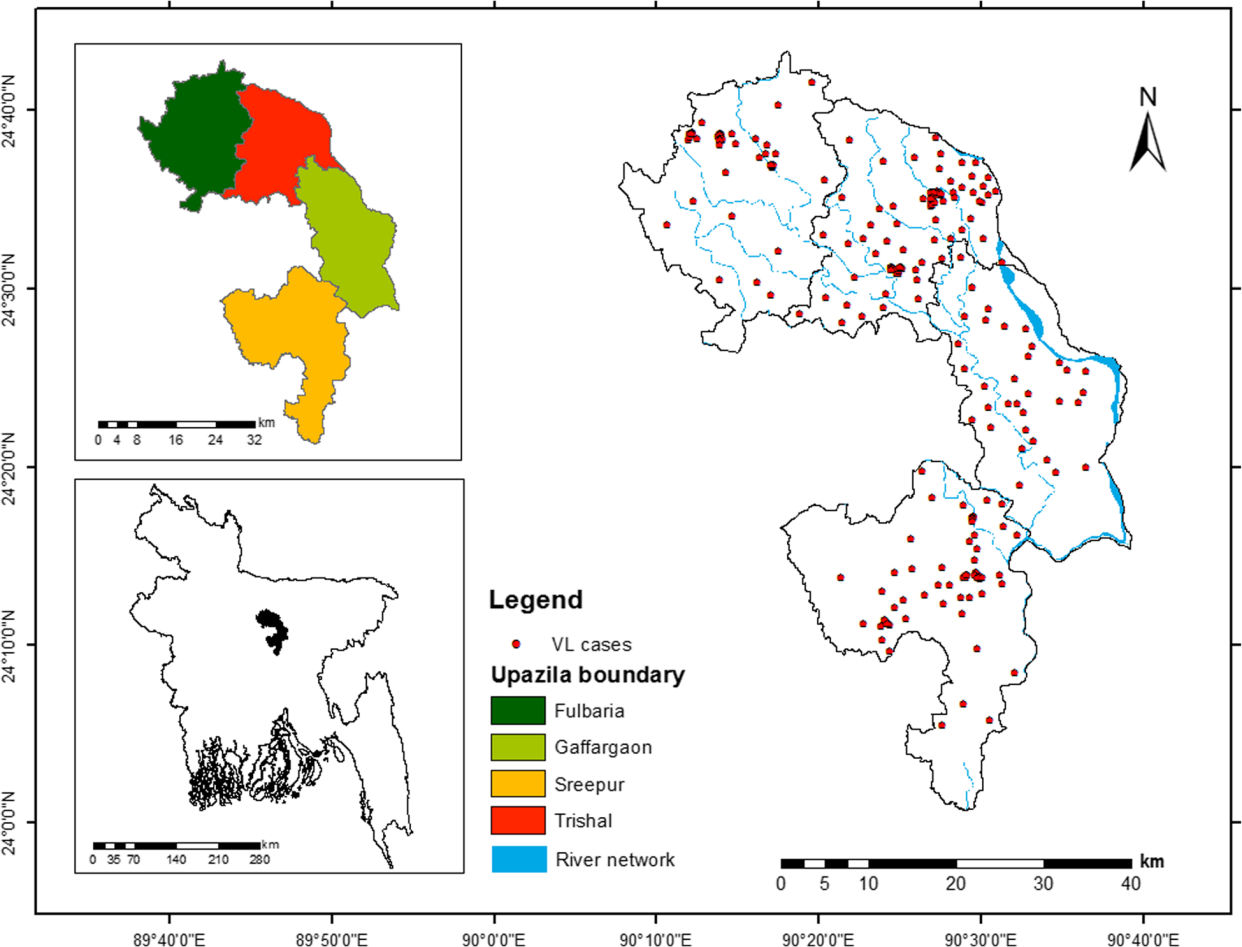
Location of the study area. *Point shapes* indicate the observed human VL cases obtained from the fieldwork

In this study, we concentrated on two districts of Bangladesh: one is hyperendemic, and the other is relatively endemic [58]. Three *upazilas* (subdistricts), namely Fulbaria, Trishal, and Gaffargaon, in Mymensingh district and Sreepur *upazila* in Gazipur district constitute the study area, which lies between longitudes of 90.26°–90.54° E and latitudes of 24.19°–24.62° N (Fig. 1). Of the 15,850 VL cases reported in Bangladesh between 2008 and 2014, Fulbaria had 4858 cases (30.65%), Trishal had 4670 cases (29.47%), Gaffargaon had 1426 cases (9.0%), and Sreepur had 283 cases (1.8%) [59]. These four *upazilas* comprised 70.92% of the total number of VL cases reported in Bangladesh. Therefore, we believe the study area provides a unique opportunity to examine the correspondence between human VL cases and potential distribution of *P. argentipes*.

### Environmental variables

The environmental data (Table 1) included 19 bioclimatic (bioclim) variables, six variables derived from remotely sensed data, and four soil variables obtained from a public database. The bioclim variables were downloaded from the WorldClim website [60]. They have a nominal resolution of approximately 1 km^2^ and were developed from monthly average climate data between 1950 and 2000 using observed data [61].

**Table 1:** Environmental variables used in this study

Serial no.	Variable	Description of the variable	Source
1	Bio_1	Annual mean temperature	www.worldclim.org/current
2	Bio_2	Mean diurnal range (mean of monthly (max temp − min temp))	
3	Bio_3	Isothermality (Bio2/Bio7) (*100)	
4	Bio_4	Temperature seasonality (standard deviation*100)	
5	Bio_5	Max temperature of warmest month	
6	Bio_6	Min temperature of coldest month	
7	Bio_7	Temperature annual range (Bio5–Bio6)	
8	Bio_8	Mean temperature of wettest quarter	
9	Bio_9	Mean temperature of driest quarter	
10	Bio_10	Mean temperature of warmest quarter	
11	Bio_11	Mean temperature of coldest quarter	
12	Bio_12	Annual precipitation	
13	Bio_13	Precipitation of wettest month	
14	Bio_14	Precipitation of driest month	
15	Bio_15	Precipitation seasonality (coefficient of variation)	
16	Bio_16	Precipitation of wettest quarter	
17	Bio_17	Precipitation of driest quarter	
18	Bio_18	Precipitation of warmest quarter	
19	Bio_19	Precipitation of coldest quarter	
20	Drainage	Soil drainage	www.barc.gov.bd
21	GST	General soil type	
22	Soil moisture	Soil moisture	
23	Soil reaction	Soil reaction or soil pH	
24	LULC	Land use/land cover	Landsat images (https://earthexplorer.usgs.gov/)
25	NDVI	Normalized difference vegetation index	
26	NDWI	Normalized difference water index	
27	TWI	Topographic wetness index	
28	LST	Land surface temperature	
29	Dem	Elevation	ASTER GDEM

Six Landsat scenes, from 2010 to 2015 (11 Mar. 2010, 14 Mar. 2011, 16 Mar. 2012, 19 Mar. 2013, 30 Mar. 2014, 17 Mar. 2015), were downloaded from the USGS site [62]. Preprocessing of Landsat data included georeferencing, subsetting, and atmospheric correction [63]. A number of derivatives were then computed, including land surface temperature (LST), normalized difference vegetation index (NDVI) [64], and normalized difference water index (NDWI) [65]. A land use/land cover (LULC) map was also derived via maximum likelihood supervised classification [63, 66]. The major LULC categories were vegetation, human settlements, waterbodies, and cultivated land. To derive LST, the thermal band of each Landsat scene was converted to spectral radiance [67], which was corrected for emissivity [68]. The images were then converted to the Celsius unit. Mean annual LST, NDVI, and NDWI data were then computed from these images. A digital elevation model (DEM) was also downloaded from ASTER GDEM [62] and used to calculate the topographic wetness index (TWI) as well as elevation surface. The TWI was calculated using slope and flow accumulation data derived from DEM [69].

Apart from the bioclim and satellite-derived parameters, edaphic layers were retrieved from Bangladesh Agricultural Research Council (BARC) [70]. All the layers were then clipped to the study area and resampled to the same geographic extent. As the bioclim variables have a nominal resolution of 1 km^2^, the Landsat-derived products and other variables were resampled to the same spatial resolution.

### Human VL cases

Multitemporal human VL cases were obtained from kala-azar patient registry logbooks located in each *Upazila* Health Complex (UHC). The *union* (lowest administrative unit in Bangladesh) and village names were then extracted from logbooks against the *mauza* (village and *mauza* are synonymous but *mauza* is used in Bengali) names, obtained from a community series database provided by the Bangladesh Bureau of Statistics (BBS). This operation resulted in a total of 3671 cases from 2010 to 2015, whose residence was within the study area. In early 2016 (January–May), we conducted several field visits to obtain the geographic coordinates of infected VL cases, occurred between 2010 and 2015, and successfully located 333 households. A Global Positioning System (GPS) was used to obtain the precise coordinates of the individual households. Two handheld GPSs (Trimble Nomad 800GXE) were employed to record the absolute location of a case. If a household had multiple cases, we recorded only one point for that household to facilitate the dispersed distribution of cases over the area of interest. Because living with or near a person who had recently experienced VL has been identified as an important risk factor in Bangladesh [9, 49], we hypothesized that the use of multiple cases from the same household could lead to household clustering that may bias our model. During the fieldwork, each household was assigned a code in the database to uniquely identify its location related to *upazila*, *mauza*, and *union*. Besides, geotagged photographs of physical and cultural features were taken to facilitate this study. The location of the 333 VL cases is shown in Fig. 1.

Given the paucity of case locations, the spatially unique human VL cases obtained from the fieldwork were used as presence data in our effort to develop an ENM. Human cases have been used as presence data in a number of previous studies to develop ENMs for determining potential vector distribution in different settings [20, 71, 72].

### Ecological niche modeling

There are two approaches to construct an ENM for modeling disease and the distribution of vectors [73]. The first approach involves modeling the species, with occurrence data, that participate in the transmission cycle [14, 16, 74] while the second approach analyzes the distribution of disease occurrence, as if it was a species, considering the entire transmission cycle and its ecological relationships [20, 71, 72]. In addition to this, *P. argentipes* has been shown to be the single proven vector of *L. donovani* in the study area [50]; thus, its distribution could reasonably be determined by the same environmental variables used to model the distribution of the disease.

In this study, we employed the second approach, using the maxent software to construct an ENM [75], which is based on a maximum entropy algorithm. This algorithm has been found to be robust in predicting vectors or species distribution from presence-only data [76, 77]. Sillero [22], however, observed that the use of presence-only data results in the identification of a “realized niche,” which is the area suitable in terms of biotic and abiotic factors. In contrast, Lobo et al. [78] noted that the use of absence or pseudo absence data increases the chance of error of estimation. In this work, we utilized field-derived VL case locations, as presence-only data to be used as input data. The maxent software computes niches by finding the distribution of probabilities closest to uniform, with the constraint that feature values match their empirical averages [79]. Based on the environmental conditions of known locations, maxent estimates a probability distribution map with a value between 0 and 1 for each cell, where 0 indicates the least suitable and 1 indicates the most suitable cell for disease or species occurrence [76]. The reasons for using maxent in this study were threefold: one, it is good at characterizing environments in a study area [80] and identifying potential niches of vectors [81]; two, the results are highly useful for regions where actual vector locations are missing or limited [76]; and three, the fact that the disease is both zoonotic and anthroponotic [82] means that a realized niche for *P. argentipes* is achievable, which is therefore a potential niche for VL and can readily become a realized niche for VL by the introduction of an infected animal or human. Hence, the second approach seems to be relevant to this work.

In this study, we used a wide range of environmental variables and developed 30 models, each with one of the 29 variables (Table 1) removed and one with none of the variables removed. The 30 models were run to evaluate the contribution of each variable to the modeling results. In each iteration, we recorded the best combination of variables by calculating the test area under the curve (AUC). High test AUC values indicate a good fit of the model to the testing data, implying high predictive power [83]. The excluded variables that caused the greatest decreases in AUC values were also recorded. This operation outputted a total of six variables, namely LST, NDWI, precipitation of the warmest quarter, precipitation seasonality, general soil type (GST), and drainage (Table 2). These variables were then used to construct an ENM to determine their influence on the likelihood of *P. argentipes* occurrence.

**Table 2:** Results of the 30 runs of the models with individual variables excluded in turn

			% Contribution in model development	Jackknife test (based on training gain)	
Excluded variable	Test AUC	Standard deviation	1st	2nd	3rd
None	0.819	0.0328	bio18	bio18	LST
Annual mean temperature (Bio_1)	0.818	0.0330	bio18	bio18	LST
Mean diurnal range (Bio_2)	0.820	0.0329	bio18	bio18	LST
Isothermality (Bio_3)	0.821	0.0327	bio18	bio18	LST
Temperature seasonality (Bio_4)	0.819	0.0331	bio18	bio18	LST
Max temperature of warmest month (Bio_5)	0.819	0.0330	bio18	bio18	NDWI
Min temperature of coldest month (Bio_6)	0.819	0.0332	bio18	bio18	LST
Temperature annual range (Bio_7)	0.820	0.0329	bio18	bio18	LST
Mean temperature of wettest quarter (Bio_8)	0.819	0.0329	bio18	bio18	LST
Mean temperature of driest quarter (Bio_9)	0.820	0.0328	bio18	bio18	LST
Mean temperature of warmest quarter (Bio_10)	0.819	0.0330	bio18	bio18	LST
Mean temperature of coldest quarter (Bio_11)	0.819	0.0329	bio18	bio18	LST
Annual precipitation (Bio_12)	0.820	0.0328	bio18	bio18	LST
Precipitation of wettest month (Bio_13)	0.818	0.0330	bio18	bio18	LST
Precipitation of driest month (Bio_14)	0.820	0.0325	bio18	bio18	LST
*Precipitation seasonality (Bio_15)*	*0.812*	*0.0337*	bio18	bio18	LST
Precipitation of wettest quarter (Bio_16)	0.820	0.0327	bio18	bio18	LST
Precipitation of driest quarter (Bio_17)	0.819	0.0329	bio18	bio18	LST
*Precipitation of warmest quarter (Bio_18)*	*0.814*	*0.0331*	GST	bio13	LST
Precipitation of coldest quarter (Bio_19)	0.819	0.0330	bio18	bio18	LST
*Soil drainage*	*0.814*	*0.033*	bio18	bio18	GST
*General soil type (GST)*	*0.811*	*0.0342*	bio18	bio18	LST
Soil moisture	0.820	0.0327	bio18	bio18	LST
Soil reaction	0.820	0.0327	bio18	bio18	NDWI
Land use/land cover category (LULC)	0.820	0.0328	bio18	bio18	LST
Normalized difference vegetation index (NDVI)	0.822	0.0321	bio18	bio18	NDWI
*Normalized difference water index (NDWI)*	*0.813*	*0.0334*	bio18	bio18	LST
Topographic wetness index (TWI)	0.819	0.0328	bio18	bio18	LST
*Land surface temperature (LST)*	*0.813*	*0.0328*	bio18	bio18	NDWI
Elevation	0.820	0.0327	bio18	bio18	LST

Italicized variables are considered to have contributed most to the model development

Out of the 333 VL case locations, 75% (248 presence points) were assigned randomly as training data and the remaining 25% (82 points) were used as testing points for model validation. Note that three duplicate points were removed by maxent during the model building. Both threshold-dependent and threshold-independent approaches were employed to evaluate the ENM. The AUC of the receiver operating characteristic (ROC) is a threshold-independent method that was used to calculate the total AUC of the sensitivity versus the fractional predicted area [77]. Models with AUC values from 0.75 to 0.90 are considered very useful, and >0.90 is considered highly accurate [72, 84]. The threshold-dependent measure was based on minimum training presence and used a one-tail binomial test. The null hypothesis was that the ENM does not predict the test/predicted presence points better than a random model. If the null hypothesis is rejected, then the ENM is a better predictor [14, 77].

To determine the variables that contributed most to the ENM construction, the percent contribution and the jackknife test of variable importance were used. The maxent algorithm measures the drop in the training AUC during permutation and expresses it as the percent contribution to the model. On the other hand, during the jackknife test, the model is developed using only one variable to find the increase in training gain and then with all variables while excluding that variable to determine the decrease in training gain [85, 86]. The variable that causes the highest gain when used in isolation encompasses the most useful information, and the one causing the highest decrease in gain contains the most unique information [83]. Therefore, we isolated the top three variables during the final ENM development based on the highest percent contribution and the two variables that produced the highest increase and decrease in training gain (when included and excluded alone). However, when a single variable was found in all three criteria, we extended our search based on the highest values of training gain, test gain, and test AUC in the jackknife test. The variables that produced the highest gains and test AUCs when used alone were considered as the most important variables [83]. The probability distribution map produced by maxent was then consulted to extract logistic probability values of each *mazua*, in order to determine suitable and unsuitable locations (based on the probability values) for the distribution of *P. argentipes*. A threshold value of 0.5 is commonly used [87] to identify moderate to high probable locations of the disease, consequently the vectors. The mean VL cases obtained from the UHC records were subsequently computed for each *mauza* and compared with the probability value. This comparison may be useful to understand the strength of the ENM developed here and the suitability of the disease distribution and, consequently, potential distribution of vectors in the study area.

## Results

The results of 30 runs of the models, where each variable was excluded one at a time and once with all the variables included, are shown in Table 2. Only six variables produced test AUC values below 0.815, namely precipitation seasonality, precipitation during the warmest quarter, drainage, general soil type, NDWI, and LST. These variables were considered to have contributed most to the model development.

**Fig. 2:**
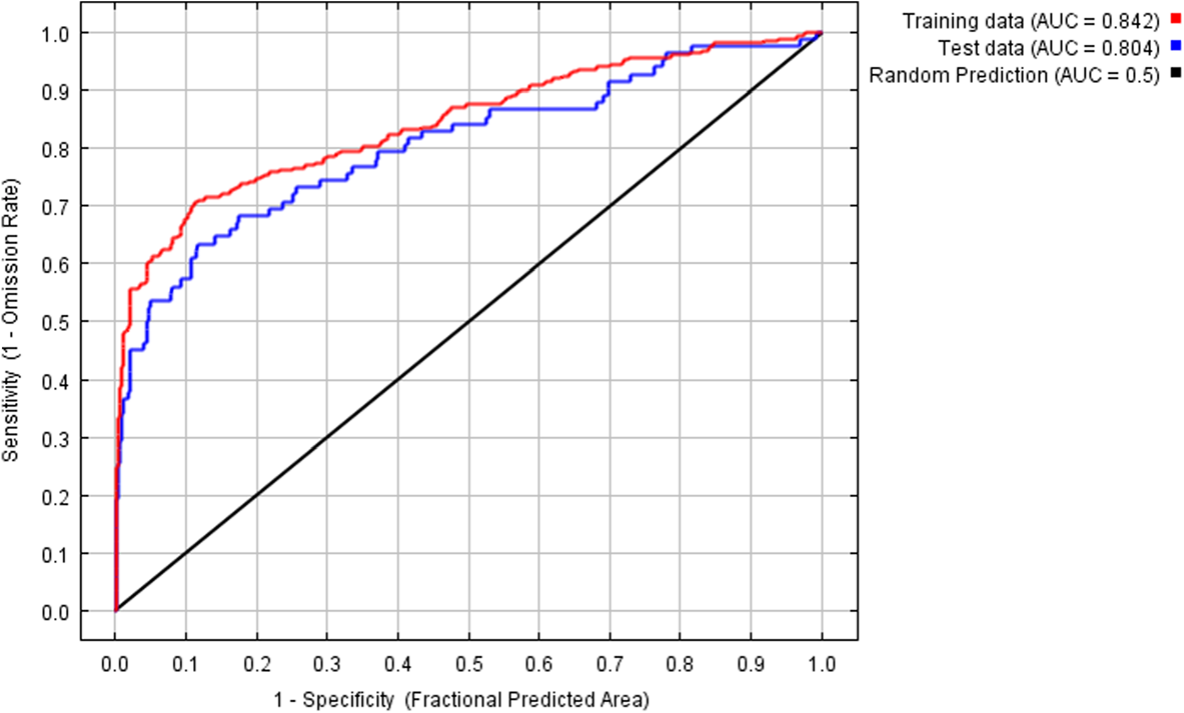
Sensitivity versus specificity graphs for VL disease distribution using the final ENM

In Fig. 2, sensitivity versus the fractional area graphs for the final ENM with LST, NDWI, precipitation of the warmest quarter, precipitation seasonality, general soil type, and drainage are shown for the training and test data. The AUC value for the training was 0.842 and the test AUC was 0.804 with a standard deviation of 0.030, indicating the performance of the model was highly satisfactory. Further, the test AUC was well above the random prediction AUC, suggesting that the ENM was much better at predicting the distribution of disease, hence the occurrence of *P. argentipes* than a random model. The narrow range of the standard deviation showed the high accuracy of the average output produced. The minimum training presence for a training point was 0.024; therefore, this value was used as the threshold in the model evaluation. The fractional predicted area was 0.977 with a test omission rate of 0.012. At this threshold, the null hypothesis was rejected because the constructed ENM performed significantly better than a random model (*p* < 0.05).

**Fig. 3:**
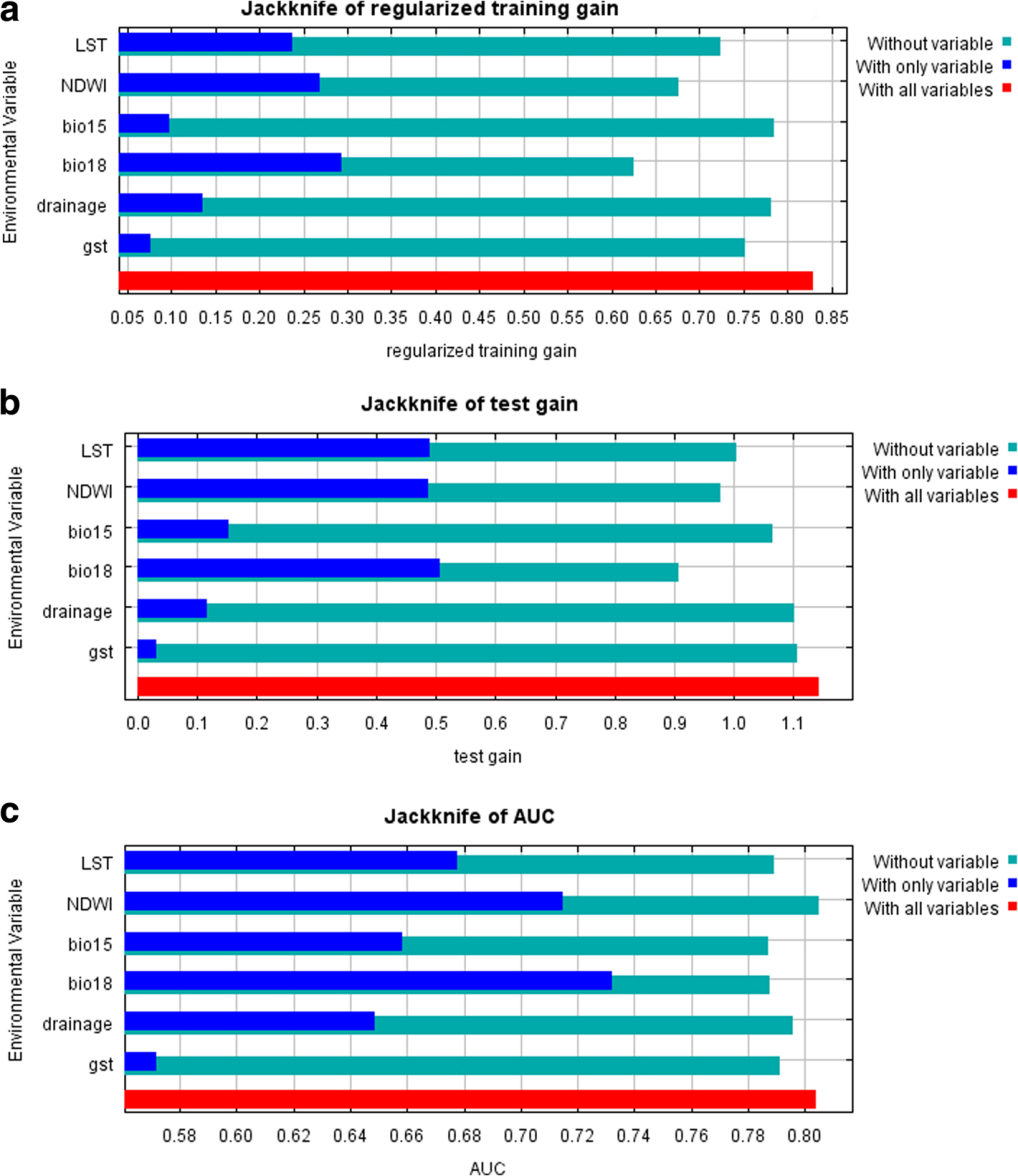
Jackknife test of variable importance in the ENM. **a** Training gain. **b** Test gain. **c** Test AUC

Table 3 shows that precipitation of the warmest quarter had the highest percent contribution, along with the highest increase and decrease in training gain in the jackknife test. As shown in Fig. 3, the jackknife test of variable importance revealed that both the test gain and test AUC were higher for LST, precipitation of the warmest quarter, and NDWI. These three variables had a total contribution of 70.2% to the model building (Table 3) and were possibly highly influential for disease occurrence. The specific probability values of these three variables showed that precipitation of the warmest quarter, which had a value of 1040 mm, was potentially suitable, while NDWI values from −0.35 to −0.10 and LSTs between 29.86 and 31.19 °C were estimated to be conducive. An LST of 29.9 °C and NDWI of −0.225 produced the highest probability of affecting the distribution of disease.

**Table 3:** Identification of the top three variables based on percent contribution, and training and test gains

Variable	Percent contribution	Training gain (without variable)	Training gain (only variable)	Test gain (without variable)	Test gain (only variable)	Test AUC (without variable)	Test AUC (only variable)
LST	15.8	0.7247	0.2379	1.0048	0.4909	0.7891	0.6779
NDWI	22.8	0.676	0.2681	0.9771	0.4878	0.8048	0.7146
Precipitation seasonality	5.6	0.785	0.0975	1.066	0.1523	0.7873	0.6582
Precipitation of the warmest quarter	31.6	0.6261	0.293	0.9063	0.5081	0.7878	0.7322
Drainage	15.4	0.7819	0.135	1.1004	0.1171	0.7958	0.6489
GST	8.8	0.7527	0.0771	1.1058	0.0326	0.7914	0.572

**Fig. 4:**
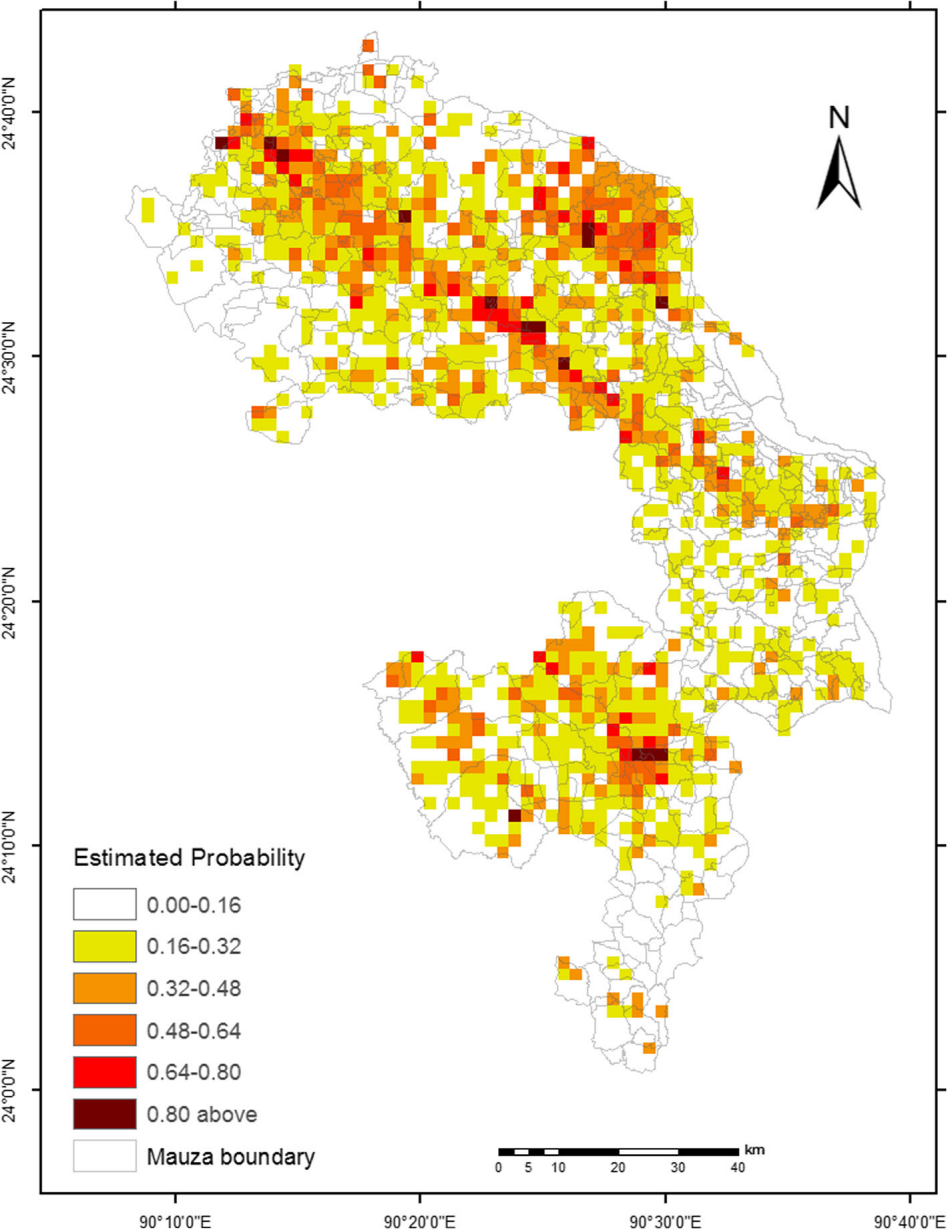
Estimated probability of the disease in the study area

The probability distribution map of the disease produced by the ENM is shown in Fig. 4. The distribution map can be used to identify areas with high and low portability of the disease occurrence or potential of *P. argentipes* distribution at the *mauza* level. Intersection of this map with the *mauza* database indicated that out of 479 *mauzas*, four in Fulbaria, three in Gaffargaon, eight in Trishal, and seven in Sreepur *upazilas* exhibited moderate to high probability of disease occurrence (Table 4), consequently the potential of the distribution of *P. argentipes* in the study area.

**Table 4:** *Mauzas* showing moderate to high probabilities of the VL cases, caused by *P. argentipes* with corresponding mean value of environmental variables

					LST	NDWI	Precipitation of the warmest quarter
	Union	Mauza	Mean cases	Probability	Mean (°C)	Mean	Mean (mm)
Fulbaria	Kushmail	Kushmail	24	0.92	30.174	−0.244	1040.833
	Achim Patuli	Achim Patuli	18	0.65	30.223	−0.221	1027.950
	Putijana	Putijana	16	0.76	29.956	−0.276	1040.833
	Radhakanai	Radhakanai Dhurdhuri	14	0.51	30.135	−0.238	1050.540
Trishal	Rampur	Kakchar	41	0.97	30.135	−0.252	1064.750
	Kanihari	Kanihari	35	0.96	30.214	−0.232	1076.440
	Harirampur	Magurjora	29	0.96	30.364	−0.211	1044.500
	Kanihari	Barma	16	0.87	30.128	−0.233	1068.800
	Harirampur	Rargaon	13	0.81	30.211	−0.233	1041.643
	Trishal	Satra Para	10	0.88	30.343	−0.237	1045.500
	Bali Para	Bahadurpur	7	0.58	30.177	−0.245	1066.667
	Kanthal	Kanthal	6	0.54	30.041	−0.248	1073.609
Gafargaon	Raona	Raona	7	0.63	30.071	−0.250	1037.667
	Jessora	Jasara	5	0.60	30.295	−0.226	1046.636
	Gaffargaon	Autbaria	6	0.52	30.105	−0.253	1050.500
Sreepur	Maona	Maona	33	0.86	30.635	−0.223	990.160
	Maona	Bara Rathura	13	0.76	30.677	−0.231	986.286
	Ward No-04	Bhangahati	12	0.80	30.457	−0.217	995.500
	Barmi	Satkhamair	9	0.71	30.478	−0.248	1004.485
	Telihati	Tengra	9	0.64	30.532	−0.229	1002.200
	Rajabari	Noagaon	8	0.66	30.366	−0.240	976.670
	Ward No-01	Ujilaba Purbapara	4	0.51	30.183	−0.288	997.000

Interestingly, of the 22 *mauzas* (27.5% of the study area), which were identified using a cutoff value of 0.5, 18 had the higher numbers of mean VL cases. This result suggests that the ENM performed well and demonstrates the correspondence between known VL cases and potential distribution of vectors. In other words, if the probability of the disease occurrence itself is being modeled, one can expect that its incidence will follow the same. In addition, the ecological requirements of the disease as a result of *P. argentipes* abundance in these *mauzas* (Table 4) indicated positive relationships between observed human cases and precipitation during the warmest quarter of the year, LST, and NDWI. For instance, the mean LST (30.17 °C), mean NDWI (−0.24), and mean precipitation of the warmest quarter (1040.8 mm) were found to account for the high VL occurrence in Kushmail *mauza* in Fulbaria *upazila*, as exemplified by the high probability of disease distribution*.* A somewhat similar association was found in Kakchar *mazua* in Trishal *upazila*. However, this association was not always true for every *mauza* investigated in this study. For instance, Achim Patuli *mauza* in Fulbaria *upazila* showed some non-linearity between mean VL cases and probability of the disease distribution.

## Discussion

VL is a life-threatening disease that disproportionately affects the poorest subset of populations in the Indian subcontinent and elsewhere [8]. We developed an ENM to demonstrate the correspondence between human VL cases and potential distribution of *P. argentipes* in endemic areas of Bangladesh. Ecological requirements of the disease occurrence caused by the sand fly vector were also determined. The analysis revealed that precipitation during the warmest quarter of the year (bio18), NDWI, and LST were the main environmental factors associated with the distribution and incidence of the disease. As *P. argentipes* is the single proven vector of *L. donovani* in the study area [50], its distribution is likely be determined by the same environmental variables. One highly likely explanation is the role of these environmental factors in regulating microclimates of the region, which potentially affect vector populations and the reservoir hosts.

Precipitation during the warmest quarter of the year appeared to be the main climatic factor influencing actual and probable incidences of the disease, which reinforces the general consensus that moist conditions are suitable for sand flies such as *P. argentipes*. High seasonality in the occurrence of the disease has been observed during the warm months in Bangladesh [9]. Therefore, the influence of precipitation during this period is not surprising as sand fly populations and the proportion of gravid females are at their highest during this period [34]. The Irrigation Support Project for Asia and the Near East (ISPAN) [88] observed that adult sand flies usually emerge during early summer (March–May) in Bangladesh and continue to thrive until the monsoon period (June–September/October) as a result of increased humidity. It is, thus, reasonable to assume that moist conditions resulting from heavy rainfall lead to the occurrence of the disease and possibly support sand fly emergence and abundance, especially in the study area, because high humidity is a prerequisite for their survival [38, 89]. Humidity determines the extrinsic incubation and vector life cycle, and Bhunia et al. [90], for example, observed that the abundance of *P. argentipes* in Bihar was associated with high humidity and heavy rainfall. Our results differ slightly from those of similar studies in Iran [91], India [39, 89], and Sudan [38] in which annual rainfall, rather than precipitation during a certain period, was reported as an influential factor affecting the distribution of the disease. The difference between our findings and those of others may stem from local physiographic and climatic characteristics of the study sites as well as differences in the methods used. Because the transmission of many parasitic diseases is confined to the summer season, changes in the lengths of rainy and dry seasons, together with changes in the intervals between seasons as a result of climatic changes, are likely to affect larvae and adult vector development and abundance [92]. Additional study is therefore warranted.

The NDWI is another important variable affecting the occurrence of the disease and potentially accountable for vector abundance in the study area. In an NDWI image derived from Landsat, negative values are a reflection of water features [65]. Over the study area, we found that water features ranged from −0.351 to 0.104, which may have contributed to the occurrence of *P. argentipes*. The presence of waterbodies, such as swamps, ponds, ephemeral canals, and marshy lands, is considered to provide suitable vector breeding sites [93]. This result suggests that people living close to waterbodies may have elevated risk of infection. Case–control studies in India confirmed that rural households in close proximity to waterbodies were at greater risk of VL than those apart [40, 94]. Bhunia et al. [39, 90] examined the effect of waterbodies/NDWI derived from satellite data on VL cases and reported a strong association, which agrees with our finding. Their study further revealed that VL cases decreased with increased distance from waterbodies. In a recent study, Abedi-Astaneh et al. [95] showed that low-lying land provides a good ecological niche for the sand fly vector. Our study area is characterized by lowlands with widespread water features; therefore, higher transmission potential of *P. argentipes* is very likely. The role of waterbodies in supporting vector abundance in the study area may be because they contribute significantly to maintaining moisture in the soil/subsoil, which suits the breeding and propagation of the immature stages of sand flies as well as provides resting habitats for the vector in adjoining domestic biotopes [40].

In addition to the presence of waterbodies in endemic areas, modification of the natural hydrological system through flood control, drainage, and/or irrigation may be equally responsible for the distribution of disease via enhanced vector activity. This was evidenced by Emch [53], who noticed that the incidence of VL cases in flood-controlled (e.g., embanked) areas of northwest Bangladesh was significantly higher than that in non-embanked areas, which he attributed to lack of seasonal flooding within the embanked areas. Despite the mechanism being not fully clear, ISPAN [88] argued that floods can wash away organic matter and larvae sites, and this process is impeded by flood control works that evidently facilitate larval development and breeding of sand flies. The efficacy of flooding on immature stages of sand fly was also observed by Mukhopadhyay et al. [96] in India, suggesting that flooding beneficially removes vector breeding sites. Hence, environmental changes caused by population growth, agricultural intensification, and development activities (e.g., flood control works, road construction) may have resulted in an environment that is humid, a factor essential for sand fly larval development. In addition, widespread rainfall during summer in the study area could lead to artificial logging and/or riverine pools due to its low elevation, which appear to affect the ecological environment of the vectors [40]. Consequently, the population dynamics of disease-carrying insects or their breeding periods and reproduction may be affected, because the breeding success of sand flies is highly dependent on rainfall duration [97]. Furthermore, people in rural Bangladesh commonly interact with waterbodies for various reasons such as fishing, bathing, and/or irrigation. These types of activities may enhance transmission potential of VL to humans. The transmission dynamics of VL in relation to waterbodies, therefore, remains a very promising area for detailed investigation.

One of the most important factors in the distribution of sand fly is temperature because it affects its survival and the speed of development of the different stages in the life cycle [98]. A temperature range of 7–37 °C with a relative humidity of >70% is highly favorable for sand fly survival in India [99]. Our analysis indicated that mean annual LST in the range 29 to 31 °C was an important predictor of the distribution of the disease, which may be useful to understand the distribution of *P. argentipes* in the study area, since one can expect that the incidence of disease generally follows the occurrence of sand fly [49]. This finding supports an earlier observation by Kesari et al. [100], who demonstrated that mean and maximum LSTs were significantly associated with sand fly density in India. Thomson et al. [101] also found that mean annual maximum daily LST from 34 to 38 °C was one of the most important ecological determinants of *P. orientalis* distribution in Sudan. Similarly, Gebre-Michael et al. [4] reported that, in East Africa, seasonal and annual LSTs were one of the best ecological determinants of *P. martini* and *P. orientalis*. Another study in Sudan, however, reported a negative relationship between LST and VL occurrence but confirmed the role of annual mean maximum and minimum daily atmospheric temperatures in the distribution of vectors [98]. Kesari et al. [100] indicated that a dry season LST of 22.23 °C and a wet season LST of 23–34 °C could account for *P. argentipes* distribution; however, in our study, we found that an annual LST of 29–31 °C likely provided a suitable niche for the incidence of the disease, suggesting the potential of *P. argentipes* survival and abundance in the study area. Nevertheless, our study together with findings from previous work in India and East Africa clearly underscored the role of LST in the occurrence of the disease.

If temperatures increase and rainfall regimes change as a result of climate warming, there would be a significant effect on vector populations. Rainfall helps to increase relative humidity, and alterations of temperature could support longevity of the vector and transmission of the disease [102]. Therefore, the impact of probable climate changes on the spread of the disease caused by sand flies (e.g., *P. argentipes*) remains a very important research topic in South Asia and Bangladesh.

Based on the probability value of 0.5, our ENM differentiated *mauzas* that had moderate to high probability of the disease (Table 4, Fig. 4), suggesting the likelihood of vector presence over space. These *mauzas* were distributed in all four *upazilas* in the study area, and in these *mauzas*, the observed mean VL cases appeared to follow the likelihood of *P. argentipes* distribution. Although the human cases in this study were retrospective, this finding does not seem to have occurred at random, meaning that our modeling approach clearly coincides the potential vector distribution with observed VL cases. This finding is partially consistent with those of Bern et al. [9] and Chowdhury et al. [11], who found that Fulbaria and Trishal in Bangladesh were highly endemic to VL. Note that the slight disagreement between probability of disease distribution and mean VL cases of three *mauzas* (Achim Patulia in Fulbaria, Satra Para in Trishal, and Naogaon in Sreepur) may have resulted from the socioeconomic situation and cultural behavior of the inhabitants of the investigated *upazilas*. An ongoing study is expected to clarify this.

A number of improvements to this study are possible. First, ENMs, such as the one developed in this study, are based on limited numbers of historical human VL cases. Small samples of occurrence points could lead to sampling bias, which may affect the overall results [25], though maxent arguably produces accurate predictions [76]. Second, inclusion of absence points (if available) could help to identify reasons for unsuitable ecological conditions, though Ortega-Huerta and Peterson [103] have suggested that this issue has a negligible effect when the spatial scale of analysis is small. Third, ENM predictions are based on input variables that affect larval development and vector survival [16]. Since we used the location of human cases as an input variable, the modeling result may be interpreted as a proxy for the potential distribution of *P. argentipes.* Fourth, the use of too many environmental variables may lead to misrepresentations due to overfitting [104], which might have increased the model complexity [77]. Fifth, we used a grid-based model to predict potential *P. argentipes* distribution based on human cases and environmental parameters as opposed to using biogeographic limits [15]. This could be another limitation. Since *P. argentipes* is the single proven vector accountable for disease transmission in the study area [50], this work did not consider other species that may be responsible for the transmission of VL in the study area. This might be another shortcoming of this work. Finally, VL case data obtained from UHCs were very poorly recorded and required very careful assimilation. Due to a shortage of funds and the sparse distribution of VL-affected households, we had to rely on a small sample size to develop the ENM. Future studies should include more samples from the endemic areas.

Despite the limitations described above, the major strengths of this study are the identification of a fine-scale regional map that shows potential distribution of the disease and the isolation of the factors affecting the occurrence of *P. argentipes*. Our modeling result could certainly be used as a proxy for potential distribution of vectors that influences sand fly distribution in the endemic areas of Bangladesh. We believe that this work may be of considerable value, especially since a recent study strongly emphasized the need for microenvironmental studies of factors that influence VL distribution in endemic areas of Bangladesh [56].

## Conclusions

To the best of our knowledge, this is the first attempt to identify underlying environmental factors that are accountable for visceral leishmaniasis (VL) incidence in Bangladesh. We developed an ENM to model the distribution of the disease which can be used as a proxy for potential distribution of vectors in endemic areas. In addition, ecological niches of the disease were identified, based on a wide range of environmental variables.

The analysis demonstrated that precipitation during the warmest quarter, land surface temperature (LST), and normalized difference water index (NDWI) were the main environmental factors influencing the occurrence of the disease in the study area. The result of the ENM may also be used to determine potential distribution of *P. argentipes*, as it is the only proven vector in the study area. Further, the occurrence of the vectors is likely to be determined by these environmental variables. This model could be used as a stepping stone to improve vector surveillance and sand fly control efforts, which could then support mapping of at-risk households. This work can be used as a baseline to model how the disease as well as vector distribution might change in the event of global environmental changes. Awareness should be raised in regions with high probability of the disease occurrence to prevent its insurgence/resurgence since it is anthroponotic in and around Bangladesh. This ecological study could be used with other socioeconomic parameters to develop a holistic and foolproof policy for eradicating the disease. We believe that knowledge of the environmental requirements to predict the distribution of the disease and, consequently, the vectors over space could help to support targeted responses to tackle this lethal disease, which affects the poorest people in Bangladesh.

## Abbreviations

AUC: Area under the curve
DEM: Digital elevation model
ENM: Ecological niche model
GPS: Global Positioning System
GST: General soil type
LST: Land surface temperature
NDVI: Normalized difference vegetation index
NDWI: Normalized difference water index
TWI: Topographic wetness index
VL: Visceral leishmaniasis
